# The invisible emotional labour sustaining LGBTQIA+ programmes within health organisations

**DOI:** 10.1371/journal.pgph.0006595

**Published:** 2026-06-24

**Authors:** Aiswarya Sasikala, Parth Sharma, Harikeerthan Raghuram, Anant Bhan

**Affiliations:** Initiative for Health Equity Advocacy and Research, Sangath, Bhopal, Madhya Pradesh, India; Independent Community Health Consultant, INDIA

## Abstract

This commentary explores the invisible identity-based emotional labour carried out by LGBTQIA+ staff coordinating community-focused programmes within civil society organisations (CSOs) that increasingly supplement public health systems in low- and middle-income countries. Drawing on lived experience and observations from LGBTQIA + –led and LGBTQIA + –affirmative health systems work in India within CSOs, we examine how LGBTQIA+ staff navigate the demands of professionalism, representation, and funder expectations within rigid institutional hierarchies. We offer recommendations for organisations and funders to recognise emotional labour and move towards ethical partnerships and supportive workplace environments.

## Introduction: Organisational Ethics without recognition of emotional labour is incomplete

In low- and middle-income countries (LMICs), government-run public health systems are supplemented by privately-run civil society organisations (CSOs). CSOs often perform core system functions, such as training medical educators and healthcare providers in public and private institutions, conducting research on public health emergencies and marginalised populations, and designing community-responsive interventions where state systems are absent, inaccessible, inadequate or not yet available.

Over the past few decades, CSOs have often become the first point of contact and the de-facto health system intermediaries for LGBTQIA+ communities to meet their health needs, especially in the wake of the HIV epidemic through funder-driven programmes. Many such programmes are coordinated by LGBTQIA+ staff, often in middle-management roles.

We use the term ‘staff’ as an umbrella term to encompass people in the diverse and precarious nature of work within CSOs. While some LGBTQIA+ individuals are hired as formal employees with protections such as staff benefits and insurance, their tenure is dictated by project-linked, rolling contracts that offer little protection or certainty of renewal once a specific grant cycle ends. Many others are engaged through even more precarious funding mandated structures as consultants, project advisors, or collaborators. These latter roles often lack any benefits and security, despite the individuals performing core system-strengthening functions. By focusing on ‘staff,’ we highlight a professionalized yet vulnerable tier of the sector that must navigate the friction between funder compliance and community needs, regardless of their specific contract type.

It is important to acknowledge that the LGBTQIA+ staff discussed in this essay often occupy a position of relative privilege compared to most of the community members (especially those with multiple marginalized identities) who face systemic barriers to housing, education, healthcare and employment.

LGBTQIA+ staff work simultaneously for their communities and within a larger ecosystem of funders, (CSO) employers, and decision-makers who shape their work but may not care for or be accountable to them. Funders often demand one-off, maximised-output activities when in fact social change and trust building require repetition, continuity, and care [1–4]. This essay examines how funder-driven health and development ecosystems systematically rely on the emotional labour of LGBTQIA+ staff, and why the recognition of this labour must be recognised as a key systems governance issue.

To understand this, we identify three categories of Civil Society Organisations (CSOs):

- Mainstream, multi-thematic CSOs: These are large organizations that have historically focused on public health and are now increasingly incorporating LGBTQIA+ health as a programmatic vertical. They are often led by cisgender-heterosexual professionals and focus on research or multi-site implementation. For staff here, the primary struggle is navigating a non-LGBTQIA+ hierarchy that may not care about political solidarity.
- Large LGBTQIA + -led CSOs: These are formally registered, community-led organizations that might have started as collectives but have scaled up to manage significant funding. In these LGBTQIA + -led spaces, emotional labour is often intensified as staff simultaneously carry community expectations, movement ethics, funder compliance, and organisational survival.
- Small LGBTQIA + -led CSOs: They are often unregistered collectives that focus on direct service provision and advocacy. They operate with the highest degree of community trust but the lowest degree of financial security, sometimes relying on the personal pockets and time of their members to sustain life-saving crisis interventions.

### Emotional and identity-based labour in routine work‌‌

Emotional labour is a requirement in many community-facing work. It is the labour involved in the regulation of emotions—inner feelings and outward expressions—to fulfil organisational interpersonal role expectations [5]. It is performed through two primary strategies: *surface acting*, where an individual modifies outward expressions to meet organizational “display rules” despite their inner feelings (e.g., faking a smile while feeling frustrated), and *deep acting*, where an individual exerts regulatory effort to modify their inner feelings to truly feel what the organization expects of them (e.g., forcing oneself to feel genuine gratitude for a short-term, precarious grant) [6].

For LGBTQIA+ staff in health and development programmes often operative within CSO contexts, these shifts happen multiple times a day: in conversations with colleagues, community members, policymakers, funders, and other interest holders. They switch codes, regulate visibility, and adapt language depending on the expectations of the listener. While this is often labelled as ‘adaptability’ or ‘professionalism’, it is in fact continuous emotional labour—labour that goes unrecognised, unpaid, and invisibilized instead of being rightfully recognised as ethical, emotional, and identity-based. For staff from systematically excluded communities, this involves the additional burden of educating others about their identities while enduring micro-aggressions or invalidations. Because emotional labour is a finite resource, the exhaustion frequently “goes home” with the employee, manifesting in chronic stress, anxiety, and sleep disruptions [7].

This labour is often dictated by frontstage/backstage acting: staff members frequently suppress anger, grief, or self-expression in frontstage institutional spaces to maintain a ‘professional’ and ‘neutral’ affect, while only being able to process these emotions in backstage community spaces [8]. While backstage spaces like online groups or queer friendships offer vital care, they are held by the same communities navigating systemic harm. Staff ultimately return to frontstage environments where this labour remains not only unrecognised but felt entirely alone.

LGBTQIA+ health and development programmes often operate as voluntary safety nets, filling the gaps left by a neglectful or weak state through temporary and often underfunded projects. In doing so, they perpetuate the **N**on-Profit Industrial Complex—a system where non-profits are tasked with managing the social consequences of inequities while leaving its root causes intact. Within this industrial complex, neutrality and politeness are rewarded over openness and resistance [9,10]**.**

While some may argue that this is part of ‘professionalism’, we must recognise that in contexts like India, ideas of professionalism are shaped by class, oppressor caste and cisgender-heterosexual norms of morality and decency. These norms, therefore, disproportionately disadvantage staff who are LGBTQIA + /Dalit/Bahujan/Adivasi/persons with disability and whose English proficiency or accents may not fit elite standards. These intersectional realities make some employees more vulnerable to scrutiny, censure or dismissal than others.

Consequently, the very entry into the health and development sector is dependent on exclusionary benchmarks such as English language proficiency, graduate education, and existing social capital within development networks. The ‘professionalization’ of these roles demands a specific set of technical fluencies such as navigating increasingly digitalised workspaces using Zoom, Microsoft Office and G-Suite, to budgeting and reporting. Because these skills are often the products of historical access to higher education and urban networks, their use as benchmarks for hiring excludes LGBTQIA+ individuals from marginalized caste, class, and regional backgrounds.

For those who gain entry, current wage structures and honoraria in the sector are often disconnected from the lived economic realities of marginalized communities. For LGBTQIA+ staff, the cost of living is often higher due to the need for private mental health support, the high cost of securing safe housing after migrating for work, reduced financial and non-financial support from natal families, lack of any financial safety bubble and savings and the reality that most institutional insurance policies explicitly exclude their partners. When wages are calculated based on ‘market rates’ that assume cis-heteronormative family structures and social safety nets, they effectively under-compensate LGBTQIA+ labour. Staff are frequently trapped in lower salary grades with no pathway for promotion, regardless of their ‘expertise by experience’ as opportunities for higher education is limited by systemic discrimination—another glaring example of systemic barriers that sustain the Non-Profit Industrial Complex.

Many smaller LGBTQIA + -led CSOs depend on state-funded public health ‘Targeted Intervention’ projects. In these projects, the health system relies on a ‘peer-led’ model that effectively exploits the lived experience of LGBTQIA+ staff while maintaining abysmally low salary caps. Peer counsellors and outreach workers are expected to perform crisis management and service delivery for as little as ₹9,000 to ₹12,000 per month, while social workers and program managers are capped at ₹18,000–₹22,000 that have not kept pace with inflation and cost of living.

Within mainstream, multi-thematic CSOs, people from LGBTQIA+ communities are intentionally recruited when a new project or vertical focused on LGBTQIA+ health is introduced. This is done in line with the emerging ‘Lived Experience’ and ‘Expert by Experience (EBE)’ frameworks. However, what is often forgotten in such spaces and processes is that the Lived Experience of LGBTQIA+ communities is heterogeneous, and heavily influenced by class, caste, disability and geography. Yet individuals recruited for projects under EBE frameworks are often those with institutional privilege, generational caste and class capital and extensive “professional” experience. These individuals then represent a palatable, sanitized and often manufactured narrative of lived experience as they fulfil various funder requirements, equity measures or organizational strategies of community engagement. This co-optation of lived experience erases those that are and have been incarcerated, unhoused, serve as political prisoners, those engaged as sex workers, and the elderly given the lack of funding/ publishing opportunities at these intersections. At the same time, it creates an environment where LGBTQIA+ Community members are expected to assimilate into existing institutional norms and project assumptions or risk erasure entirely [11,12].

This further begs the question: what differentiates a LGBTQIA+ person in a lived experience role as opposed to their work as staff in CSOs? The answer often lies in politics of identity and the lack of epistemic agency afforded to the most affected in defining lived experience‌‌.

In all these settings, LGBTQIA+ staff routinely negotiate their presence and identities in the workplace by suppressing discomfort, deflecting ignorant or insensitive remarks, neutralising one’s politics in meetings, internal or email communication and documentations and enduring queerphobic comments while maintaining a ‘professional’ and ‘neutral’ affect. These negotiations occur in the context of interactions both within the organisations (with colleagues in other departments, administration, finance and leadership) and within the wider health and development system actors (other CSOs, collaborators, funders and policymakers). When LGBTQIA+ staff are visibly queer, they risk tokenisation or invasive questions; when they are not, they risk being in spaces where prejudice goes unchallenged. They must regulate their attire, language, and self-presentation in response to organisational and funder expectations.

This unrecognised emotional labour is shared by staff with intersectional lived experience—for example, someone who is Dalit or Neurodivergent—where the pattern of harm is consistent: staff feel accountable to everyone but cared for by no one. Dismantling such structural complicity and achieving meaningful reform requires power shifts, and institutional courage; this becomes paramount when the vision, mission and policy statements of organisations remain progressive in language but hollow in practice.

## The structural conditions producing emotional labour

Emotional labour of LGBTQIA+ staff do not occur in a vacuum. They are a product of different structural conditions: (1) the dynamics of funder relationships, (2) funding volatility in the Global North and program closures, and (3) the role of CSOs as intermediaries.

### The dynamics of funder relationships and emotional labour

In funder-driven systems, CSOs are tasked with providing services through LGBTQIA+ programmes under extractive systems that prioritise funder-defined project outcomes over community-rooted transformation. With the rise of philanthropy-funded non-profits; activists and LGBTQIA+ staff are pulled away from collective struggle into professionalised and sanitized CSO spaces, transforming political work into deliverables and measurable impact [9]. Between community ties and institutional roles, LGBTQIA+ staff are constantly pulled in multiple directions: *are we working towards what is most needed, or what is easiest to measure? And what does ethical labour mean when emotional labour is invisible*?

Even ‘progressive’ funders often set boundaries on what kind of LGBTQIA+ health program is within funding remits. That is the reason why short-term LGBTQIA+ sensitization programmes are considered fundable, whereas securing sustained funding to dismantle stigma or discrimination is difficult.

Funders’ insistence on accounting for every rupee or dollar is shaped by philanthropic, capitalist, and colonial traditions of governance where they must demonstrate efficiency and accountability to their own boards, taxpayers, or shareholders [2]. This obligation is often exercised by treating grantee CSOs as less than equal partners who require strict scrutiny and surveillance as contractors to produce measured results. In doing so, they reproduce the same logics of efficiency, accountability, and individual responsibility that underpin neoliberal markets [9].

Thus, funders always require reports and metrics as tools for evaluation and monitoring, whereas the LGBTQIA+ staff working in CSOs experience them as pressures to produce ‘success stories’ while dealing with conflict, frustration, or precarity in their programmes and work with marginalised communities. This push to prove value for money and demonstrate ‘efficiency and impact’ undermines deeper, slower processes of systemic change and community building and instead force CSOs and LGBTQIA+ staff within them to focus much of their efforts on assessment, monitoring, and reporting [13,14].

Funders often emphasise ‘collaboration’ and ‘partnership’, but the funder-grantee relationship remains deeply asymmetrical in practice. A *cold-toned phone call* from a funder, or an unexpected demand for urgent project related data or information during the early mornings, requires careful self-regulation from staff—switching codes, neutralising defensiveness, avoiding the impression of being perceived as ‘difficult’. Whereas funders can delay disbursals, change timelines, be rigid in their expectations or impose new requirements with no accountability, the staff must accept these changes, keep the communities engaged and other staff motivated despite imminent scarcity. Funding structures routinely normalise such unequal power relations, where emotional, identity-based, and administrative labour is simply assumed to exist without recognition, credit, or compensation, almost always in addition to existing program work.

The situation is further complicated in a project receiving foreign funding. India’s Foreign Contribution (Regulation) Act (FCRA) imposes stringent requirements and operational restrictions on how funds can be received and used. The FCRA requirement that strictly prohibits registered entities from sub-granting of funds is a blow to collaborative, trust-based work, which used to allow the larger CSOs to channel resources to smaller, community-rooted groups who may lack FCRA certification, and the capacity to write proposals or manage funder relations [15]. At the individual level, these regulations create a “crisis of legibility”; the prohibition against staff using personal funds (as such expenses might not be reimbursed later) for immediate needs, coupled with the requirement that every transaction be traceable to a verified bank account, is particularly exclusionary for LGBTQIA+ individuals. For those whose legal IDs or bank records do not align with their gender identity, the simple act of receiving an honorarium becomes a retraumatizing experience. LGBTQIA+ staff absorb these institutional failures, mediating between bureaucratic constraints and community accountability while carrying personal financial and emotional precarity. These are added layers of emotional labour that remain undocumented, invisible, and unsupported.

This regulatory pressure manifests internally as institutional risk-aversion, leading to further friction between the organization and the community. The layered crisis of trust is most visible during administrative friction. For example, when a finance team driven by fears of FCRA non-compliance and audits is forced to resort to raising frequent questions, it can offend funders, community members and delay crucial payments. The middle-management LGBTQIA+ staff often bears the brunt of this: they must perform *surface acting* to absorb the funder’s irritation while simultaneously explaining payment delays to the community member. In these moments, the staff member is seen as the ‘representative of the organization’ by the community member, and as the ‘community advocate’ by the organization’s finance or leadership teams. They are forced to spend emotional labour to maintain a trust that are being eroded by the funding, legal and institutional structures.

### Funding volatility in the Global North and program closures

In the backdrop of adverse funder relationships and short-term funding cycles, recent volatile political shifts in the Global North have led to funding cuts, and abrupt program closures. The February 2025 survey by the International Council of Voluntary Agencies (ICVA) notes that over 85% of NGOs are affected by the recent US funding cuts and reported either halting activities or facing major operational disruptions [16]. These funding cuts are rooted in the biopolitics of global anti-gender movements where various societal actors are actively challenging or rejecting LGBTQIA+ rights, scholarship and work, as well as supporting policy changes that erode infrastructure needed to sustain these communities [17]. Often done under the guise of protecting culture, children and religion, these transnational events are concerted efforts to occupy the bodies of LGBTQIA+ communities as a smokescreen for authoritarian political theatre [18,19].

In India, global right-wing and ‘anti-gender politics have found resonance among religious fundamentalists and conservative political actors. These actors frame the spectrum of queerness as a ‘Western imposition’ which is a threat to the traditional institutions of family, children, and national culture, while using Western-originated anti-gender language to justify legal and social exclusion [20]. These abrupt disinvestments are actively adversely impacting global health ecosystem and forcing local organisations to absorb the immediate shock while sacrificing long-term systemic resilience [21]. When projects end abruptly in such situations, LGBTQIA+ staff are left with little time to reconcile funder-mandated project closeout formalities with their ongoing ethical accountability to communities. The contract mandates that the grantee must return funds on termination of the project. But what about the return of trust and promises made to communities that are now left abruptly without services? Additionally, staff members must attempt to figure out complications around administrative procedures such as navigating the lack of a provision for the return of funds received under FCRA.

The case of Hyderabad’s unique *Mitr* Clinic, which served between 150–200 LGBTQIA+ clients each month until its sudden closure in February 2025, is an example [22]. The closure felt like a personal loss for community members, staff, and for the fragile ecosystem of LGBTQIA+ health programmes. It reopened five months later as the *Sabrang* Clinic with funding raised because of efforts by the staff and community members [23]. But such reopenings are rare. Mostly abrupt project closures mean that LGBTQIA+ staff are forced into deciding which services to cut first, which team members or community facilitators to let go, which lives to prioritise, and whether to remain silent in hopes of protecting future funding [24]. Work then begins to feel like being constantly under siege, a relentless triaging of needs and harms in the face of scarcity. And yet, the grief of LGBTQIA+ staff remains undocumented. They are expected to grieve quietly, while continuing to report professionally, thank funders for their support and to carry the hope of continuity when everyone around them fears collapse. This invisible labour extends to surviving staff who must take on additional responsibilities while processing their own uncertainty. This labour is compounded by a hierarchy of professional legitimacy. While organizational leaders often hold the privilege of being perceived as “objective professionals” by funders and other interest holders, these middle-management staff are not given the same concession. These staff members can be perceived as “too close to the issue,” and therefore expected to manage community grief and crisis quietly, ensuring that the friction of the work does not disturb the organization’s relationship with its funders. This isolates the staff member as both the representative of the community and the implementer of the funder’s rigid protocols and at the same time they navigate these tensions without the institutional power to change the policies they are expected to implement.

Beyond India, similar patterns have been documented across South Asia, South America, and East Africa. A 2025 global survey found that over half of LGBTQIA+ organisations across 59 countries reported staff layoffs or program closures [25], while a rapid assessment documented acute threats to organisational survival across Southeast Asia [26]. Across contexts, respondents emphasised the emotional toll of uncertainty and the burden of mediating between community accountability and rigid funder constraints. As development assistance retracts, funders frequently withdraw not only financial resources but also coordination and continuity functions, leaving local organisations to absorb the resulting operational and ethical pressures [27]. These findings reveal a transnational pattern of funder volatility that intensifies the emotional, administrative, and ethical labour borne by LGBTQIA+ staff, who are left to absorb the consequences of geopolitical decisions while sustaining trust, care, and professional legitimacy within their communities.

In India, this volatility has recently escalated with the Transgender Persons (Protection of Rights) Amendment Act, 2026. Under the guise of ‘protecting’ the community, the Act introduces sweeping criminal provisions that penalize ‘alluring,’ ‘compelling,’ or ‘forcing’ any person to ‘adopt a transgender identity’, with penalties extending up to life imprisonment—effectively criminalizing lifesaving peer support, gender-affirming healthcare, and community outreach work done by CBOs and CSOs [28].

### The role of civil society organisations as intermediaries

Despite the precarious landscape of funding relationships and state surveillance, CSOs occupy a critical space between funders and communities, shaping the day-to-day realities of LGBTQIA+ staff. While funders may set the rules, organisations determine how these rules get translated into practice by determining the workflow, developing policies which define what can be done and not be done, work environment and the distribution of support. As discussed earlier, the CSOs are not a homogeneous category, and their internal cultures are shaped by their leadership and history. One way in which the LGBTQIA+ led CSOs are different from the CSOs led by cis-heterosexul professionals is that the latter nudge LGBTQIA+ staff to be the ‘face of the community’ during celebratory periods like Pride Month. However, this visibility is rarely translated into power on global stages and this enthusiasm rarely translates into solidarity during moments of political crisis. When the state actively betrays its LGBTQIA+ citizens through anti-rights legislation or discriminatory policies, this non-partisan institutional voice often falls silent. This silence is experienced by LGBTQIA+ staff as a form of abandonment. It forces staff to maintain a ‘professional’ facade and continue routine work while their legal personhood is being debated.

CSOs have the relative power to determine whether to shield their staff from extractive donor demands or pass those demands down as administrative mandates. All institutions—CSOs or otherwise—are responsible for creating ethical and conducive workspace climates that align with human values and not just the program values. This means establishing internal mechanisms for care and redress, ensuring that reporting demands do not compound stress, and fostering cultures where LGBTQIA+ staff are not isolated in their roles as both representatives and implementers. If institutions can become co-creators of ethical programmes, they can help the LGBTQIA+ staff thrive by helping translate funder intent into practices that uphold care, trust, and sustainability. Yet, care and ethics are offered as one-time workshops on work-life balance, resilience, and self-care or endearing verbal platitudes rather than being treated as organisational responsibilities. Staff are encouraged to manage burnout individually instead of questioning the systemic conditions that cause such chronic fatigue.

For staff working across multiple projects, especially during periods of increased funding precarity, these dynamics extend into their personal livelihoods. Many calculate their own salaries, negotiate extensions, and figure out employment continuity across grants. This individualisation of responsibility to survive precarity reflects neoliberal organisational logics, even when institutions benefit from staff flexibility.

## Towards ethical partnerships

Moving LGBTQIA+ programmes beyond procedural ethics requires acknowledging the invisible labour carried by staff. Current structures and practices place the moral burden almost entirely on the staff, who must stretch limited resources, absorb emotional costs, and manage abrupt closures with little support. Making funders and CSOs accountable as key interest holders for the relational and emotional climate they create through their practices is crucial.

Those feminist and trust-based funders who prioritize relational accountability, and multi-year core funding, are experienced as a rare form of kindness. This reveals how unusual it is in funding relationships to be met with thoughtfulness, reciprocity, or genuine care. However, such funders are still in short supply and are often oversubscribed, with grantees driving into highly competitive application processes where they must demonstrate that they deserve the funding to sustain the vital work.

Emerging frameworks such as participatory grant making, trust-based philanthropy, feminist funding, and decolonial approaches positions funders as ethical actors within the system. Such funders call for relationships rooted in transparency, collaboration, and reciprocity [29]. Applied in LGBTQIA+ health program contexts, these approaches would mean seeing LGBTQIA+ staff not merely as tokens of representation and as dispensable labour, but as ethical partners in shaping, evaluating, and sustaining programmes. The funder's responsibility thus does not end with disbursing money and enforcing accountability; it would mean standing with institutions, staff, and communities in moments of uncertainty, grief, and transition.

Funders and CSOs could consider the following recommendations towards a more just future:

- Ethical partnerships begin with funding structures that recognise time, trust, and relational work as core to LGBTQIA+ programming. Hence, funding must move to multi-year, flexible, and core funding that enable sustainability and prioritise long-term community wellbeing over short-term outputs.
- Direct and flexible funding must be made available to LGBTQIA+ collectives that often remain unregistered because of lack of resources or for safety, autonomy, and political reasons. Strengthening smaller collectives decentralises decision-making and avoids extractive relationships that draw on lived experience.
- Accessible proposal formats, proposal-writing support, and low-burden dialogic reporting must be provided to the grantees. They can foster mutual learning while making emotional and relational labour visible.
- During instability, funders and CSOs must share responsibility through bridging support, advance communication, and transparent decision-making. Without this, the emotional labour of “guessing” funder expectations or absorbing sudden shocks will fall on LGBTQIA+ staff
- Compensation and wage for LGBTQIA+ staff must account for the higher costs for safe housing and access to private, affirming healthcare. Organizations must formalize pathways for staff sustainability with equitable opportunities to gain leadership roles based on ‘expertise by experience.’CSOs must move beyond individualised self-care and establish collective mechanisms for equity and safety, including stronger institutional ethics committees, reflective supervision, and regular policy audits. Fair and timely pay, transparent salary structures, and explicit recognition of identity-based labour are essential to reducing precarity.
- CSOs must adjust project plans to include time for community building, crisis management, travel fatigue, and relational work. This would require leadership to be trained on identity-based labour, caste and other forms of identity dynamics, and relational ethics to help them make such equitable decisions.
- At a systems level, funders and organisations must engage with policy circles and relevant decision makers for enabling regulatory environments, including reconsidering policies such as India’s FCRA sub-granting prohibition, and redesign funder processes to work ethically within existing constraints.
